# Enhancement of the catalytic efficiency and thermostability of *S* *tenotrophomonas* sp. keratinase KerSMD by domain exchange with KerSMF

**DOI:** 10.1111/1751-7915.12300

**Published:** 2015-11-10

**Authors:** Zhen Fang, Juan Zhang, Baihong Liu, Guocheng Du, Jian Chen

**Affiliations:** ^1^ Key Laboratory of Industrial BiotechnologyMinistry of EducationJiangnan UniversityWuxi214122China; ^2^ Synergetic Innovation Center of Food Safety and NutritionJiangnan UniversityWuxi214122China; ^3^ School of BiotechnologyJiangnan UniversityWuxi214122China; ^4^ Key Laboratory of Carbohydrate Chemistry and BiotechnologyMinistry of EducationJiangnan UniversityWuxi214122China; ^5^ National Engineering Laboratory for Cereal Fermentation TechnologyJiangnan UniversityWuxi214122China

## Abstract

In this study, we enhanced the catalytic efficiency and thermostability of keratinase KerSMD by replacing its N/C‐terminal domains with those from a homologous protease, KerSMF, to degrade feather waste. Replacement of the N‐terminal domain generated a mutant protein with more than twofold increased catalytic activity towards casein. Replacement of the C‐terminal domain obviously improved keratinolytic activity and increased the *k_cat_*/*K_m_* value on a synthetic peptide, succinyl‐Ala‐Ala‐Pro‐Phe‐*p‐*nitroanilide, by 54.5%. Replacement of both the N‐ and C‐terminal domains generated a more stable mutant protein, with a *T* *_m_* value of 64.60 ± 0.65°C and a half‐life of 244.6 ± 2 min at 60°C, while deletion of the C‐terminal domain from KerSMD or KerSMF resulted in mutant proteins exhibiting high activity under mesophilic conditions. These findings indicate that the pre‐peptidase C‐terminal domain and N‐propeptide are not only important for substrate specificity, correct folding and thermostability but also support the ability of the enzyme to convert feather waste into feed additives.

## Introduction

Feather waste is a significant by‐product of poultry farming and contains high levels of protein (Onifade *et al*., 1998; Gupta *et al*., 2013; Daroit and Brandelli, 2014). Due to the rapid development of poultry and pig farming in China (Song *et al*., 2014), it is worth investigating the use of recycled feather waste as an inexpensive source of protein that can be added to animal feed stocks. Biodegradation by proteases is preferred, as this process results in less damage to the amino acids than degradation induced by chemical or physical digestion (Gupta and Ramnani, 2006). Compared with common proteases, such as pepsin and trypsin, keratinases are more efficient due to their unique ability to hydrolyse insoluble proteins containing disulfide bonds (Brandelli, 2008). However, recent studies have shown that the commercial utilization of keratinases is limited due to their lack of heat stability and catalytic efficiency (Brandelli, 2008; Teresa and Justyna, 2011; Gupta *et al*., 2013), i.e. the thermal inactivation or low activity of keratinases is a common problem (Gupta *et al*., 2013).

We previously showed that a newly isolated keratinase, KerSMD, from *Stenotrophomonas maltophilia* BBE11‐1 had the capacity to degrade feather waste and textile fabric (Fang *et al*., 2013). Additionally, KerSMD was successfully expressed in *Escherichia coli* and could more efficiently degrade a feather substrate than the classical keratinase KerA from *Bacillus licheniformis* (Fang *et al*., 2014). However, the thermostability and catalytic efficiency of KerSMD still need to be improved to realize its potential for commercial application. Protein engineering techniques, such as error‐prone polymerase chain reaction (PCR) and site‐directed mutagenesis, have been used to overcome the limitations of natural enzymes (Böttcher and Bornscheuer, 2010). Successful directed evolution requires extensive preparation, and knowledge of the crystal structure of an enzyme is needed for the rational design of site‐directed mutagenesis.

In this study, a simple protein‐engineering technique was used to replace the functional domains of KerSMD. From the many protein sequences available, the keratinase‐like protease KerSMF was chosen for replacing the functional domains of KerSMD. KerSMF, which has high expression efficiency and catalytic efficiency, shares more than 40% homology with KerSMD within the N/C‐terminal domains, as well as high identity (80%) in the conserved hydrophobic motifs (N1 and N2) and catalytic domains (Fang *et al*., 2014). Replacing domains from KerSMF might increase the chances of constructing successful mutants. Since the pre‐peptidase C‐terminal (PPC) domain is probably related to thermostability and substrate specificity (Fang *et al*., 2014), we hypothesized that by deleting or altering the PPC domain, we could alter the KerSMD enzyme's stability and catalytic efficiency. The catalytic efficiency of alkaline protease HP70, from *Stenotrophomonas maltophilia*, was found to be improved by truncation of the C‐terminal domain (Ribitsch *et al*., 2010; 2012). Importantly, the KerSMD/KerSMF PPC domains and N‐propeptides have almost the same molecular size and model structure, which may improve the probability of successful exchange. The PPC domain from KerSMF (T1) showed more *β*‐folds in the predicted secondary structure. We deduced that T1 probably contributed to the stability of the mature protein. To the best of our knowledge, such modifications of keratinases, by replacing PPC domains, have not previously been reported. Furthermore, the swapping of N‐terminal domains could improve the substrate specificity and thermostability of keratinases (Rajput *et al*., 2012). Although the N‐propeptides of KerSMF and KerSMD were auto‐cleaved after protein maturation, folding efficiency and structural stability could be changed by different N‐propeptides. In this study, we hypothesized that the domains from KerSMF could improve the enzymatic properties of KerSMD.

KerSMD domains were swapped with KerSMF domains to construct new variants, and deletion mutants lacking the C‐terminal domain were also constructed. There was obvious enhancement of catalytic efficiency and thermostability by domain swapping, while deleting the C‐terminus resulted in a decrease in structural stability and keratinase activity. Homology modelling of KerSMD and a C‐terminal variant was also conducted to investigate the possible mechanism by which the PPC domain enhanced catalytic efficiency on different substrates. To the best of our knowledge, this is the first study on improving the activity and heat stability of a keratinase by exchanging PPC domains, illustrating that the C‐terminus is important for thermophilic keratinase activity.

## Results

### Construction, expression and purification of recombinant proteins

**Figure 1:**
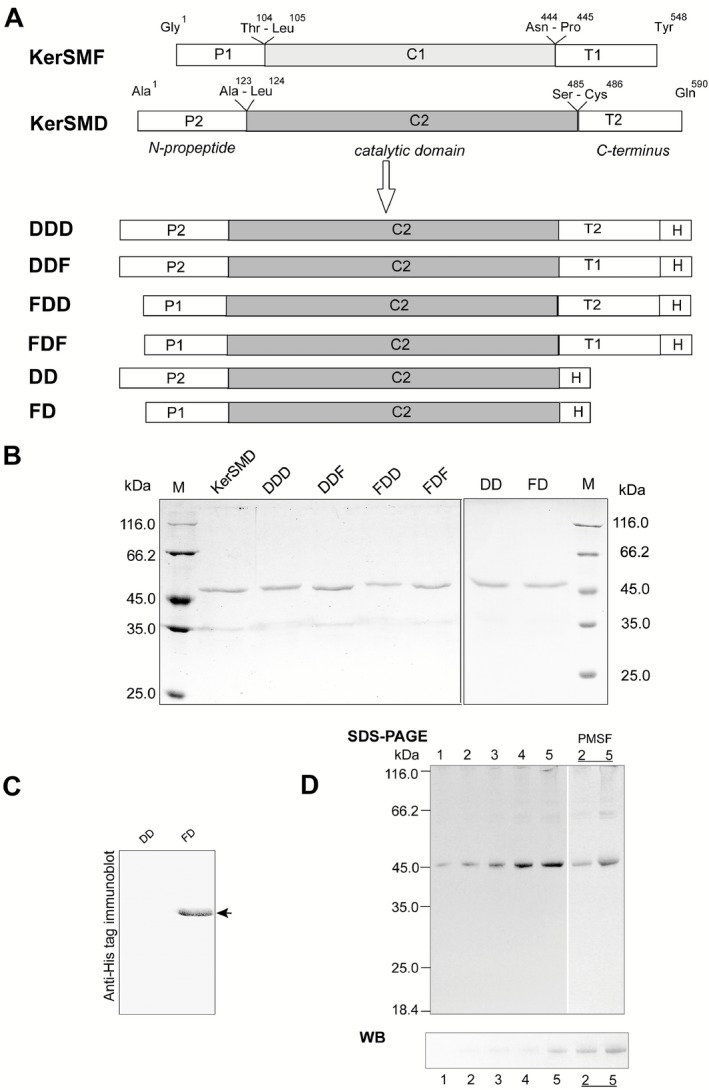
Construction, expression and purification of recombinant proteins. Schematic representation of primary protein structure (A), SDS‐PAGE analysis (B), and anti‐His tag immunoblot analysis (C and D) of recombinant KerSMD and mutant proteins, DD and FD. A. Boxes P1 and P2 represent the N‐propeptides, C1 and C2 the catalytic domains, and T1 and T2 the PPC domains. Amino acid residues at the border between two regions are labelled. H represents the fused His tag. Different combinations of boxes represent precursor proteins. B. Purified proteins (1 μg) were analysed by SDS‐PAGE for molecular size. M denotes the marker lane. C. Western blot analysis of C‐terminal deletion mutants (DD and FD) using an anti‐His tag antibody. Arrows indicate the positions of the His tagged target proteins. D. Different amounts (1, 2, 3, 4 and 5 μg) of DD were analysed by SDS‐PAGE and Western blot (WB). Before being subjected to SDS‐PAGE, 5 mM PMSF was added to the precursor proteins of DD (2 μg and 5 μg) to inhibit proteolysis.

By aligning amino acid sequences of KerSMF and KerSMD (Fig. S1A), we found that they can be divided into three fragments, the N‐propeptide (P1 and P2), peptidase S8 catalytic domain (C1 and C2) and PPC domain (T1 and T2) (Fig. 1A). The homologous fragments shared different levels of sequence identity, i.e. more than 80% identity between catalytic domains C1 and C2, 34% identity between T1 and T2, and 32% identity between P1 and P2. Although the two PPC domains were of equal length, the predicted secondary structure of T1 had more *β*‐folds, which might improve protein stability (Fig. S1B). Therefore, T1 (residues P341 to Y441) was chosen to replace T2 (residues C363 to N467) to improve stability, generating the C‐terminal variant, DDF, where D designates domains from KerSMD and F a domain from KerSMF. In addition, the N‐propeptides were also exchanged to produce the novel mutant FDD. The double swap of N/C‐terminal domains generated mutant FDF. C‐terminal deletions were based on mutants FDD and DDD (KerSMD) and generated FD and DD respectively. The primary structures of all recombinant proteins are shown in Fig. 1A.

Except for DDD and DD, which were purified using Phenyl FF and Q FF, proteins were purified using a Ni‐chelating column. The molecular size of each purified mutant protein was determined by sodium dodecyl sulfate‐polyacrylamide gel electrophoresis (SDS‐PAGE) analysis (Fig. 1B). This showed that mutants DDD and FDD have approximately 46.5 kDa molecular weight, while DDF and FDF have approximately 48 kDa; since the PPC domains of KerSMD and KerSMF each contained approximately 100 amino acids, these approximate sizes were acceptable. Interestingly, the actual size of the C‐terminal deletion mutants FD and DD approached that of wild‐type keratinase (48 kDa), which was higher than the predicted value (36 kDa). Analysis by matrix‐assisted laser desorption/ionization time‐of‐flight/time‐of‐flight (MALDI‐TOF‐TOF) mass spectrometry confirmed that purified DD was derived from KerSMD (Table 1). The N‐terminal amino acids of the mature proteins, DD and FD, were sequenced, and found to be EPGA and GDVQ respectively. It seemed that the N‐propeptides were not completely auto‐processed after protein maturation, and the size of DD approached that of DDD. We deduced that the unexpected slow migration of DD and FD was related to the addition of N‐terminal domains that contributed to their high molecular weights. In order to confirm the actual size of DD and FD, Western blotting with a His tag antibody was performed using 1 μg loading samples (Fig. 1C). This showed clear immunostaining of mutant FD, close to the position of the protein band on SDS‐PAGE, while DD was not observed. Therefore, different amounts of DD (1, 2, 3, 4 and 5 μg) were used for Western blot analysis. We found that high amounts of DD (4 μg and 5 μg) showed clear immunostaining (Fig. 1D). We also tried using 5 mM phenylmethanesulfonyl fluoride (PMSF) to inhibit the autoproteolysis of preformed DD. The immunoblot results showed that DD (2 μg and 5 μg) formed an obvious band (Fig. 1D), which was close to the apparent size of FD (48 kDa). The molecular weight of the N‐propeptide of DD was predicted to be approximately 12.88 kDa, close to 11.47 kDa, which is the size of the PPC domain T2 of the mature form of DDD, consisting of the catalytic domain C2 and PPC domain T2. It was probable that the size of DD was similar to that of DDD. However, the wild‐type protein and the mutants – FDD, DDF and FDF – all shared the same N‐terminal sequence APND, and their N‐termini were correctly cleaved.

**Table 1:** MALDI‐TOF‐TOF mass spectrometry analysis of purified DD

Residues	Observed mass	Expected Mr^b^	Calculated Mr	ppm	Sequence	Ion score
382–397	1708.7841	1707.7768	1707.8363	−35	K.YRPASCDGVVTVGATR.I	89
398–411	1565.6959	1564.6886	1564.7522	−41	R.ITGGITYYSNYGTR.V	86
477–487	1142.5560	1141.5487	1141.5914	−37	K.GKDPLAPAAMR.T^a^	20

a Methionine (M) reflects the oxidation status.

b Mr means relative molecular mass.

### Enzymatic activities of different mutants

The enzymatic activity of all mutants was based on their caseinolytic and keratinolytic properties (Table 2). With the swapped N‐propeptide P1, the FDD mutant showed a significant increase in caseinolytic activity, of at least twofold, compared with the activity of the wild‐type (3779 U mg^−1^). The keratinolytic activity was most affected by swapping or deletion of PPC domains. The T1 domain allowed DDF to achieve the maximum keratinolytic activity, 3909 U mg^−1^, while deletion of the T1 domain resulted in a substantial decline of DD enzymatic activity on a feather substrate, to only 448 U mg^−1^. The double‐displacement strategy that generated FDF decreased keratinolytic activity but helped improve caseinolytic activity.

**Table 2:** Enzymatic activity of different mutants with casein and feather meal substrates

Enzyme	Activity with different substrates (U mg^−1^)	
	Casein	Feather meal
DDD	3779 ± 20	3409 ± 56
DDF	4467 ± 14	3909 ± 45
FDD	7844 ± 25	3453 ± 25
FDF	8229 ± 50	2158 ± 56
DD	4328 ± 18	448 ± 45
FD	9876 ± 50	3086 ± 20

The impact of PPC domain deficiency was determined by comparing DD and FD. The keratinolytic activity of mutant DD was negligible, while FD retained activity at 3000 U mg^−1^, higher than that of FDF, where it was fused with the PPC domain. Additionally, FD showed maximum catalytic activity (9876 U mg^−1^) on casein. It seemed that the P1 domain could replace the PPC domain to maintain keratinase activity. Since the N‐propeptide was covalently linked to the C2 domain during the catalysis reaction, substrate specificity and catalytic efficiency probably depend on the type of N‐propeptide. We concluded that P1 helps mature enzymes to increase their caseinolytic activity while minimally affecting their keratinolytic activity.

### Thermostability of different mutant enzymes

**Figure 2:**
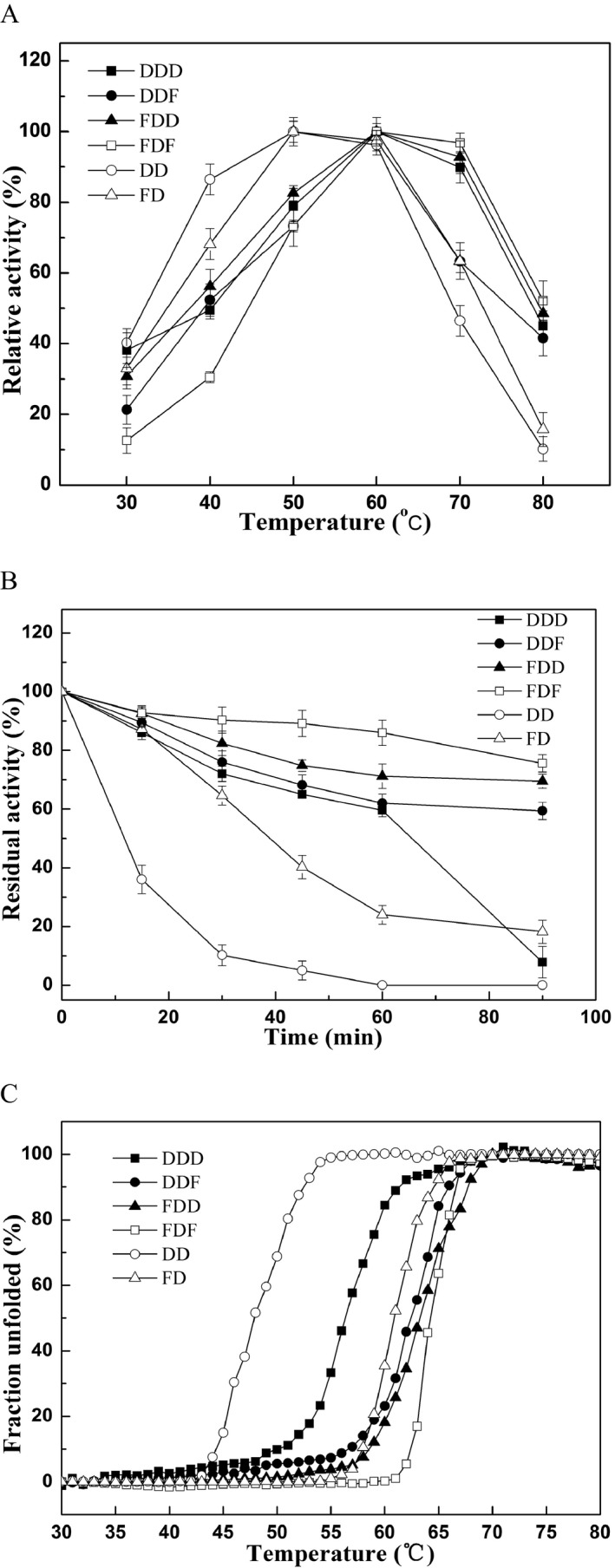
Thermal stability of KerSMD (DDD), DDF, FDD, FDF, DD and FD mutant proteins. (A) Effects of different temperatures on enzyme activity. (B) Enzyme activity at 60°C after different time intervals. (C) Thermal denaturation of different mutants. The change in CD value at 222 nm with increase in temperature was recorded to produce these curves. The Y‐axis represents the percentage of unfolded protein. The initial CD values of untreated enzymes were regarded as 0%, and the CD values of fully denatured enzymes were regarded as 100%.

The displacement or deletion of N/C‐terminal domains not only affected the substrate specificity but also changed the thermostability of the enzyme. It seemed that the fusion of PPC domains increased the thermophilic properties of KerSMD. As shown in Fig. 2A, the temperature for maximum activity of the wild‐type (DDD), DDF, FDD and FDF was 60°C. Furthermore, these mutants (DDF, FDD and FDF) with fused PPC domains were thermophilic, showing more than 60% activity when heated for 90 min at 60°C (Fig. 2B). FDF was the most stable mutant with a *T_m_* value of 64.6°C, and maximum half‐life (*t_1/2_*) of 244.6 ± 2 min at 60°C, which was 5.9‐fold more than the wild‐type (Fig. S2). On the other hand, the C‐terminal deletion mutants, DD and FD, showed maximum activity at 50°C. From Fig. 2C and Table S1, it was clear that the *T_m_* value of DD was 8.2°C lower than that of the wild‐type. Deletion of their PPC domains widened the temperature range of the enzymes. Although the activity and thermostability of both DD and FD drastically decreased when the temperature increased above the range of 60–70°C, they still showed more than 70% relative activity at 40°C. In particular, DD showed an enhancement in enzyme activity of nearly 38% at 40°C compared with the wild‐type (Fig. 2A).

### Alkaline stability of different mutants

The effects of alkaline pH on enzyme activity and stability were also studied. Although all mutants retained the catalytic domain C2, obvious differences were observed with respect to alkaline stability, as shown in Fig. S3. The wild‐type protein and mutants showed maximum activity under alkaline conditions (pH 8.0 to 9.0). Notably, the introduction of C‐terminal domain T1 contributed to improved activity of DDF and FDF at pH 10.0. It also contributed to the optimal alkaline stability of FDF, which showed an improvement of 12% compared with the wild‐type. DDF, with the fused T1 domain, also showed improved stability at pH 9.0–12.0. On the other hand, the deletion of PPC domains in DD and FD resulted in a substantial decrease in alkaline stability, but widened the pH range of DD and FD towards acidic conditions (Fig. S3A). FD was the most unstable mutant under alkaline conditions, but still retained 40% activity at pH 12.0 and nearly 90% relative activity at pH 6.0, which was an approximately 30% improvement compared with the wild‐type (Fig. S3B). The covalently linked N‐propeptide P1 might play a key role in improving FD activity in an acidic environment, compared with a weak effect of the P2 domain. The data showed that covalently linked P1 could rearrange the distribution of electrons in catalytic groups. Displacing the C‐terminal domain helped keratinases to adapt to high pH, and the P1 domain could broaden the application of enzymes in both acidic and alkaline environments.

### Kinetic parameters of wild‐type and mutant keratinases in connection with various synthetic peptides

Since small synthetic peptides could bind to the catalytic centre and would help elucidate various properties of the S1 pocket, we determined the catalytic efficiency of various synthetic peptides, namely AAPF, AAPL and AAVA (Table 3). We found that these three peptides have different residues at P1 and P2 sites, which contain hydrophobic amino acids. The catalytic efficiency determinant, *k_cat_*/*K_m_*, reflected the difference in enzyme activity compared with the wild‐type, while the *K_m_* value represented the substrate‐binding ability. When using the synthetic peptide AAPF as the substrate, only FDD, FDF and FD mutants showed an obvious decrease in catalytic efficiency compared with the wild‐type. Although we observed a higher *K_m_* value with the AAPF substrate that indicated a decrease in substrate‐binding ability, all mutants showed an obvious increase in catalytic efficiency with the AAVA substrate. The average *k_cat_*/*K_m_* value with AAVA was the lowest observed, while substrate‐binding ability (1/*K_m_*) was high for all three mutants. The hydrophobicity at position P1 for the three synthetic peptides was in the following order: AAPF > AAPL > AAVA. It seems that the catalytic efficiency and substrate‐binding ability of keratinase are related to the hydrophobic side‐chain of its peptide substrates.

**Table 3:** Kinetic parameters of wild‐type KerSMD and various mutants

Substrate	Enzyme	*K_m_* (mM)	*k_cat_* (s^−1^)	*k_cat_*/*K_m_* (s^−1^ mM^−1^)	Ratio^a^
AAPF	DDD	0.66 ± 0.04	46.0 ± 0.4	71.0 ± 5.2	0.0%
	DDF	0.30 ± 0.01	33.0 ± 0.1	109.0 ± 0.2	+54.5%
	FDD	1.80 ± 0.09	23.0 ± 0.2	13.2 ± 0.5	−81.7%
	FDF	2.20 ± 0.09	25.0 ± 0.6	11.0 ± 0.2	−84.5%
	DD	0.73 ± 0.02	94.2 ± 0.2	129.0 ± 3.9	+81.7%
	FD	2.60 ± 0.05	20.0 ± 0.1	7.9 ± 0.2	−90.1%
AAPL	DDD	0.98 ± 0.02	33.0 ± 2.1	33.0 ± 1.6	0.0%
	DDF	1.90 ± 0.15	65.4 ± 2.7	33.0 ± 1.2	0.0%
	FDD	1.30 ± 0.07	60.0 ± 0.4	48.0 ± 2.4	+45.5%
	FDF	1.50 ± 0.02	65.2 ± 0.2	43.2 ± 0.8	+30.3%
	DD	1.90 ± 0.08	88.0 ± 3.8	47.0 ± 0.1	+42.4%
	FD	2.60 ± 0.04	87.1 ± 0.5	33.0 ± 0.7	0.0%
AAVA	DDD	0.49 ± 0.01	2.20 ± 0.03	4.41 ± 0.12	0.0%
	DDF	0.56 ± 0.03	2.90 ± 0.13	5.10 ± 0.10	+15.9%
	FDD	0.72 ± 0.02	4.40 ± 0.02	6.08 ± 0.20	+38.6%
	FDF	0.43 ± 0.03	3.00 ± 0.34	7.03 ± 0.51	+59.1%
	DD	0.64 ± 0.03	4.50 ± 0.40	7.02 ± 0.30	+59.1%
	FD	0.53 ± 0.02	3.00 ± 0.03	5.50 ± 0.20	+25.0%

a The percentage increase or decrease in catalytic efficiency (*k_cat_*/*K_m_*) relative to the wild‐type (DDD).

When using AAPF as the substrate, replacing the T2 domain with T1 to generate DDF obviously improved catalytic efficiency, resulting in the highest *k_cat_*/*K_m_* value of 109 s^−1^ mM^−1^. The lowest *K_m_* value, which was the main contribution to optimal catalytic efficiency, indicated that the PPC domain was probably an important factor for substrate binding. This was confirmed by the contrary results obtained with C‐terminal deletion in mutants FD and DD, which showed lower binding affinity for AAPF than the wild‐type. We also found that deletion of the PPC domain might be helpful in increasing catalytic efficiency, especially in the DD mutant. The results suggest that the PPC domain increased substrate‐binding ability but reduced catalytic efficiency when AAPF was used as the substrate. When the N‐propeptide was swapped to the P1 domain, mutants FDD and FDF preferentially degraded the AAPL substrate. AAPF and AAPL, with strongly hydrophobic amino acids and long side‐chains, might have some properties that are similar to those of feathers' proteins, which mainly consist of large hydrophobic amino acids, such as Phe and Leu.

### Secondary structure assay and modelling of DDD and DDF

**Figure 3:**
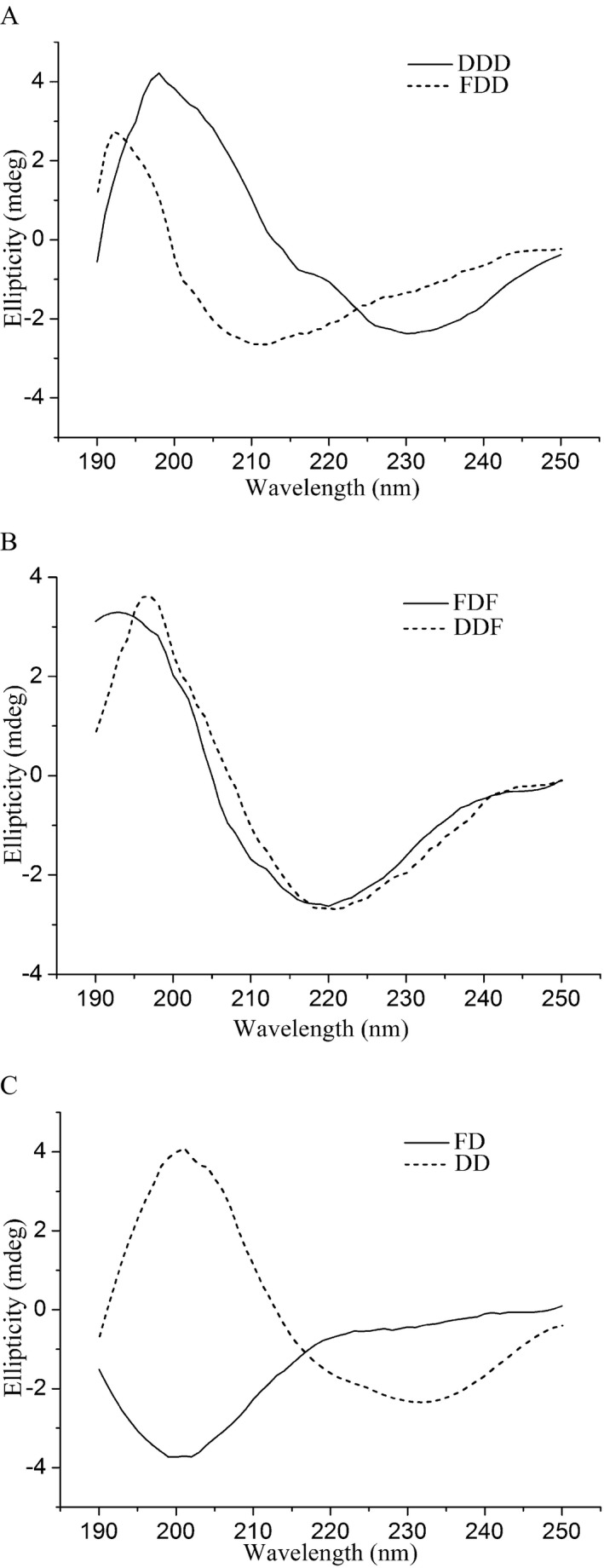
Far‐UV CD spectra of different mutant proteins. Mutants with a similar mature form were compared. (A) DDD and FDD. (B) FDF and DDF. (C) FD and DD. CD spectra were measured at 25°C.

Before structural modelling, a secondary structure assay was performed to obtain information for a reliable model. Far‐UV circular dichroism (CD) spectra of DDD and the mutants DDF, FDD, FDF, DD and FD were determined (Fig. 3). The CD curve of FDD drifted to the left and was obviously different from that of DDD (Fig. 3A). As shown in Table S2, DichroWeb analysis showed that DDD had a larger percentage (29.0%) of unordered coils than FDD (21.2%). It seemed that the N‐propeptide P1 reduced the amount of unordered structure during the protein‐folding process, which resulted in enhancement of structural stability.

**Figure 4:**
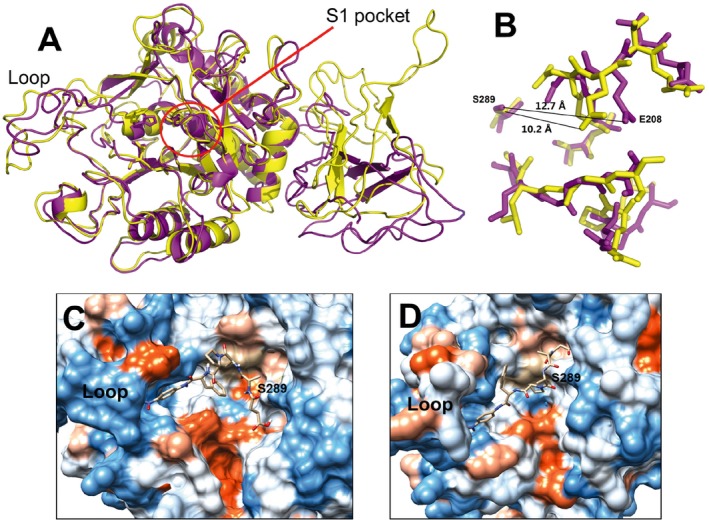
Homology models of keratinases DDD and DDF. Models were generated using the modeller 9.11, PyMOL molecular graphics system and namd software for energy minimization. (A) Alignment between the DDD (yellow) and DDF (purple) structures. The catalytic centre, composed of D42, H105 and S289, is represented by sticks. The S1 pocket is highlighted with a red circle. The position of the exosite loop (D85–D101) is indicated. (B) Alignment of the residues of the S1 pockets of DDD (yellow) and DDF (purple), which are formed by residues 176–180, 204–210 and 215–218. The distance between S289 and E208 is indicated by black lines. (C and D) Hydrophobicity surface around the S1 pockets of DDD (C) and DDF (D). The hydrophobicity increases from blue to orange. The succinyl‐AAPF‐*p*‐nitroanilide peptide stick structure is modelled onto the substrate‐binding pocket. chimera v1.9 (www.cgl.ucsf.edu/chimera/download.html) was used to compute the hydrophobicity, and AutoDock was used for docking. Residue S289 and the exosite loop are indicated.

Since the P1 and P2 N‐propeptides showed low amino acid identity (< 25%) based on blast results from the Protein Data Bank (PDB) database, they were not considered for modelling. However, the T1 and T2 PPC domains shared high identity (31% and 50%) with the C‐terminal domain of *Vibrio* extracellular metalloprotease (vEP C‐ter 100; PDB no. 2LUW), which suggested the possibility of constructing homology models of the mature forms of DDD and DDF. These two model structures were aligned as shown in Fig. 4A, with DDD representing wild‐type keratinase. Based on the model alignment, we could appreciate that DDD had a higher proportion of unordered loops, which was also confirmed by the secondary structure (Table S2). In contrast to the PPC domains, which mostly comprised turns and unordered loops, flexible loops were present in the catalytic domain, which was the major distinction between DDD and DDF. Thus, we speculated that the PPC domain affected the flexibility of loops.

The S1 pocket was predicted as per BprV protease (PDB no. 3TI9), which shared 49% identity to the catalytic domain C2 (Wong *et al*., 2011). Therefore, we represented residues 176–180, 204–210 and 215–218 as constituting the S1 pocket in our homology models of keratinases DDD and DDF. Although the two mature proteins showed the same catalytic domain, C2, slight differences were observed, mainly outside the S1 pocket as well as in the exosite loop (D85–D101), affecting the substrate binding and catalytic efficiency. As shown in Fig. 4B, the distance between E208 and S289 was increased by 2.5 Å due to the influence of PPC domain T1, in which S289 was the catalytic residue. This change might expand the size of the S1 pocket, enabling it to receive AAPF or macromolecular substrates, such as keratin. In addition, the more hydrophobic loop above the S1 pocket in DDF could affect its ability to accommodate an insoluble substrate (Fig. 4C,D). Therefore, docking with the synthetic peptide AAPF was performed to identify the location of substrate‐binding sites. AAPF was bent to suit the limited volume of the S1 pocket of DDD (Fig. 4C); this may have reduced the access of AAPF to the hydrolysis reaction. Contrarily, DDF, which has a larger volume of the S1 pocket, bound to the linear AAPF substrate, thus avoiding any obstruction of the space. This was confirmed by the low *K_m_* value of DDF (Table 3), suggesting that the T1 PPC domain changed the stereochemical structure of the S1 pocket to influence the substrate‐binding efficiency of DDF, as well as its alkaline stability.

## Discussion

### Role of PPC domains

The keratinases KerSMD and KerSMF both had a distinct extension (of about 100 amino acids) at their C‐terminus and shared high homology, whereas the PPC domain is rarely seen among the common keratinases from *Bacillus*, *Streptomyces* and *Pseudomonas* sp. (Lin *et al*., 1995; 2009; Gupta and Ramnani, 2006; Li *et al*., 2007; Gupta *et al*., 2013). In this study, the PPC domain showed important roles in keratinase maturation, structural stability and catalytic activity.

PPC domains are related to keratinase maturation, where the PPC domain may take part in activation and then be cleaved off after secretion of the peptidase (Yeats *et al*., 2003; Zhao *et al*., 2008). Recent reports stated that C‐terminal deletion contributes to improve expression and polypeptide catalysis rate of the extracellular serine proteases from *S. maltophilia* (Ribitsch *et al*., 2010; 2012). The N‐propeptides of subtilisin‐like proteases, even the short N‐terminal prosequence of intracellular subtilisin protease, are cleaved during protein maturation (Li *et al*., 1995; Gamble *et al*., 2011; Shinde and Thomas, 2011; Dai *et al*., 2012). However, in the present study, we found that the C‐terminal deletion mutants DD and FD were covalently linked to the N‐propeptide after maturation. This unique phenomenon indicates that the PPC domain in KerSMD possibly leads the N‐propeptide towards the active site for cleavage. Analysis of the crystal structure of the keratinolytic protease fervidolysin has shown that the N‐propeptide is additionally anchored to the PPC domain by the hydrophobic pocket at the interface and affects protein maturation (Kim *et al*., 2004). In this study, we deduce that the interaction between the N‐propeptide and PPC domain of *Stenotrophomonas* sp. keratinase is similar to that in fervidolysin, while more evidence is required for confirmation. The use of a propeptide in structural studies may broaden our knowledge of protein folding.

The independent PPC domain had the function of changing the substrate specificity, which has been reported for many collagenases (Zhao *et al*., 2008; Yan *et al*., 2009). In the case of KerSMD, the deletion of the PPC domains affected its catalytic efficiency as well as substrate binding to small synthetic peptide substrates, similar to the results reported by Ribitsch and colleagues (2010). Comparing the catalytic kinetics of DDD and DDF suggested that the C‐terminal domain had a steric influence on the S1 subsite around the catalytic centre. Our homology model might be helpful in inferring the direct or indirect influence of the PPC domain; it showed that using PPC domain T1 increased the volume of the S1 pocket such that it could accept a larger and hydrophobic substrate, such as AAPF. We also observed that the PPC domain could improve enzymatic activity on a macromolecular substrate, namely feathers. It seemed that the PPC domains of KerSMD and KerSMF were similar to the C‐terminal domain of vEP, which was essential for degrading insoluble proteins (Yun *et al*., 2013). Therefore, we inferred that PPC domains probably exist specific sites on the keratinase surface to bind the feather substrate.

In addition, the function of the PPC domain of enhancing the structural and alkaline stability of the keratinase should not be ignored. From the comparison of predicted secondary structures (Fig. S1B and C) and CD spectra (Fig. 3), we deduced that increasing the number of *β*‐folds could improve the stability of mutants. It has been reported that the hyperthermostable protease Tk‐SP required a C‐terminal *β*‐jelly roll domain to retain its hyperstability (Foophow *et al*., 2010). Since the charge distributions of the PPC domains T1 and T2 are different, it seems that the T1 PPC domain could help KerSMD to adapt to high pH environments, which suggests that KerSMD could degrade feather waste at both acidic and alkaline pH. The pH value of the substrate will increase towards alkalinity due to the release of amino acids during feather degradation. Therefore, the use of basophilic and mesophilic mutants could be favourable for the energy‐saving treatment of feather waste, as well as waste from some depilation procedures (Onifade *et al*., 1998; Gupta *et al*., 2013).

### Role of N‐propeptide domains

The N‐terminal propeptides of proteases (e.g. subtilisin E and BPN′) act as intramolecular chaperones to catalyse folding and facilitate maturation (Gallagher *et al*., 1995; Shinde and Thomas, 2011; Dai *et al*., 2012), and we observed similar functions for the N‐propeptides of KerSMD and KerSMF. The exogenous N‐propeptides inhibited the activity of the mature enzyme but could refold inactive proteins that were expressed without the propeptide (Fig. S4). It is hypothesized that the P1 domain was more efficient in inhibiting enzyme activity than the P2 domain. The refolding of subtilisin BPN′ needed an intramolecular chaperone‐forming prosegment complex, which also had the ability to inhibit protease activity (Gallagher *et al*., 1995). We concluded that the inhibitory effect of the P1 N‐propeptide might be more efficient in facilitating enzyme overexpression, maturation and molecular modification due to inhibition of cytotoxicity and autohydrolysis.

The N‐propeptide played an important role in determining spatial structure and enzyme activity during enzyme maturation. By swapping N‐propeptides, the P1 propeptide reduced the proportion of unordered loops and the FDD mutant showed a change in secondary structure (Fig. 3, Table S2), which probably contributed to the increase of enzyme activity on different substrates. With the help of new N‐propeptides obtained by site‐directed mutagenesis, improvement in the refolding efficiency and enzymatic activity of subtilisin BPN′ has been achieved (Li *et al*., 1995). Selecting a suitable N‐propeptide to improve enzyme properties could be more convenient for directed evolution than site‐directed mutagenesis (Takagi and Takahashi, 2003; Rajput *et al*., 2012; Martinez *et al*., 2013). We believe that, in addition to the N‐propeptide from KerSMF in this study, suitable propeptides from other proteins will eventually be identified.

The swapping of the N‐propeptide also improved the structural stability of KerSMD. Rajput and colleagues (2012) swapped the prosequences of KerBL and KerBP to enhance thermostability and keratinolytic activity. In our study, the swapping of N‐propeptides between KerSMD and KerSMF enhanced the heat stability of KerSMD but did not give similar results for KerSMF, indicating that folding of the catalytic domain C1 was more precise. P1 decreased the proportion of unordered coils or loops in KerSMD, which might reduce the flexibility required to initiate thermal unfolding (Vihinen, 1987; Kumar *et al*., 2000; Martinez *et al*., 2013). Thus, mutant FDD acquired higher stability than the wild‐type protein. However, the covalently linked N‐propeptide was not helpful for increasing the *T_m_* value of mutants DD and FD. This indicated that the PPC domain was the main contributor to thermostability. In addition, the P1 domain improved pH stability. Since the propeptide had the function of refolding the steric structure of the catalytic centre, the polar residues might be exposed or hidden to change the charge distribution.

Surprisingly, catalytic efficiency was also affected by N‐propeptide swapping. Although limited knowledge is available to explain the underlying mechanism, it has been proposed that changes in catalytic efficiency are related to geometrical changes in the S1 pocket (Georgieva *et al*., 2001; Wong *et al*., 2011). This was partially confirmed by the kinetic parameters of different peptides and a hydrophobic surface map of the homology model (Table 3 and Fig. 4). We observed that KerSMD and its derivatives were similar to some common subtilases and keratinases that preferentially cleave Leu or Phe at the P1 position (Georgieva *et al*., 2001; Gupta *et al*., 2013). A similar protease, StmPr1 from *S. maltophilia*, which degrades insoluble collagen and fibronectin, also shows high specificity for Leu and Phe (Windhorst *et al*., 2002). Since Leu and Phe residues have long hydrophobic side‐chains and are present at a substantial level in keratin (Wong *et al*., 2011), the enhancement of catalytic efficiency on the AAPL and AAPF substrates may be related to keratinolytic activity.

The double‐displaced mutant FDF showed enhanced enzymatic properties. This indicated that protein engineering might be very helpful for extending the application of keratinase to the fields of poultry and pig farming, which require a more complete digestion of the substrate for animal consumption. Moreover, the heat and alkaline stability of FDF also highlights its potential application in the process of leather tanning, which utilizes high temperature and an alkaline environment (Gupta *et al*., 2013).

## Experimental procedures

### Reagents

Chemicals of analytical grade were purchased from Sigma‐Aldrich (Shanghai, China). *Escherichia coli* BL21 (DE3) and the plasmid pET22b were purchased from Novagen (Darmstadt, Germany). Isopropyl‐β‐D‐thiogalactoside (IPTG) and PrimeSTAR HS DNA polymerase were obtained from TaKaRa (Dalian, China). Kits for plasmid extraction and DNA purification were purchased from Sangon (Shanghai, China) or TaKaRa. Oligonucleotides were synthesized by Sangon. Restriction endonucleases were purchased from Fermentas (Shanghai, China).

### Source of genes and bacterial cultivation

The genes encoding KerSMD (GenBank KC814180) and KerSMF (GenBank KC763971) were amplified from the *Stenotrophomonas maltophilia* BBE11‐1 genome (China Center for Type Culture Collection M2012495). *Escherichia coli* BL21 (DE3) was used as the expression host and cultured in Luria–Bertani (LB) medium with 100 μg ml^−1^ ampicillin at 37°C.

### Cloning and vector construction

The KerSMD/pET22b(+) plasmid system was previously constructed by our group. All gene manipulations were performed in accordance with standard molecular cloning protocols, as previously described by Fang and colleagues (2014). In this study, KerSMD and mutants were cloned into the pET22b vectors using the NcoI and XhoI restriction sites. All mutants used the signal peptide *pelB* leader and the His tag GGFHHHHHH at the N/C‐terminus. Mutants displacing the N‐ or C‐terminal domain were constructed using overlapping PCR. The gel‐purified PCR products were digested with two restriction endonucleases, NcoI and XhoI, and ligated to the pET22b vector digested with the same restriction enzymes through incubation with T4 DNA ligase at 16°C overnight. The ligation mixture was directly transferred into competent *E. coli* BL21 (DE3) cells for verification and expression. Except for P1 and P2, which were cloned into pET28a(+), the construction of other recombinants was based on the pET22b vector system.

### Expression and purification of recombinant keratinases

*Escherichia coli* BL21 (DE3) strains harbouring plasmid pET22b and target genes were cultured in LB medium at 37°C until the OD_600_ reached 0.6, after which the temperature was reduced to 20°C and IPTG was added to a final concentration of 0.2 mM for induction. The culture was incubated for 72 h before cells were harvested by centrifugation. Expressed proteins of inclusion bodies were obtained by ultrasonication and overnight dialysis in 20 mM phosphate buffer (pH 7.4) at 4°C. Crude recombinant keratinases were purified via three columns, HiTrap^TM^ Phenyl FF, Q FF, and HisTrap^TM^ FF crude, using an AKTA purifier (GE Healthcare, Sweden). The Phenyl FF column procedure was conducted as previously described by Fang and colleagues (2014). Gradient elution of the Q FF column was performed, using 20 mM Tris‐HCl pH 8.2 (buffer A) and 1 M NaCl in 20 mM Tris‐HCl at pH 8.2 (buffer B), at a flow rate of 1 ml min^−1^. The Ni‐chelating affinity chromatography column was equilibrated with 20 mM phosphate buffer (pH 7.4) containing 20 mM imidazole and 0.5 M NaCl. Recombinant proteins were eluted with 20 mM phosphate buffer (pH 7.4) containing 0.5 M imidazole and 0.5 M NaCl. Fractions with protease activity were collected, analysed by SDS‐PAGE and stored at −80°C.

### SDS‐PAGE, Western blot analysis and protein sequencing

SDS‐PAGE was carried out as previously described by King and Laemmli (1971), and Western blot analysis was according to the procedure of Ghosh and colleagues (2009). Except for DD, which was loaded in different amounts (1–5 μg), 1 μg was loaded onto the SDS‐PAGE gel for all samples. In order to reduce the autoproteolysis of DD, 5 mM PMSF was added to two samples (2 μg and 5 μg) as a control. After SDS‐PAGE, the target proteins were blotted onto a polyvinylidene difluoride membrane (Bio‐Rad, Shanghai, China) and stained with Coomassie brilliant blue R‐250. A His tag antibody (Novagen, Shanghai, China) was used for blotting. The band of purified protein was excised for sequencing by standard Edman degradation; this was carried out by Sangon using an Applied Biosystems 492cLC protein sequencer (Thermo Fisher Scientific, Waltham, MA, USA). We also used MALDI‐TOF‐TOF mass spectrometry (Bruker Daltonics ultrafleXtreme, Bruker Daltonik GmbH, Bremen, Germany) to identify DD, according to Ribitsch and colleagues (2010).

### Keratinolytic and caseinolytic activity assays

Keratinolytic activity was tested using feather meal from a local chicken farm, while caseinolytic activity was measured using casein as the substrate at 60°C. The assays were performed as previously described by Fang and colleagues (2014) with minor modifications. One unit of enzyme activity was defined as an increase in absorbance by 0.001 AU min^−1^ ml^−1^ at 660 nm. Protein concentration was determined by using a Bradford protein assay reagent kit (Beyotime, Shanghai, China) with bovine serum albumin as the standard. All experiments were repeated three times and the standard deviation was calculated.

### Biochemical characterization of wild‐type and mutant keratinases

Keratinolytic activity of purified enzymes was recorded at different temperatures (ranging from 30°C to 80°C) in 50 mM glycine‐NaOH buffer (pH 9.0). Maximum activity was considered as 100% and the activity of various mutant enzymes was calculated as a percentage, in terms of relative activity compared with the wild‐type. The thermal stability of various enzymes was determined by incubating the samples at 60°C for different time intervals. The initial value prior to activity loss was recorded as 100%, and residual activities were expressed as a percentage of this initial value.

To determine the optimal pH for maximum enzymatic activity, several buffers at different pH values were used to incubate the purified enzymes as follows: NaAc‐HAc (pH 4.0–6.0, 100 mM), Na_2_HPO_4_‐NaH_2_PO_4_ (pH 6.0–8.0, 100 mM), Tris‐HCl (pH 8.0–9.0, 100 mM), and glycine‐NaOH (pH 9.0–12.0, 100 mM). The enzymes to be assayed were incubated in different buffers at 60°C and the pH stability was determined after overnight incubation of each enzyme dissolved in different buffers at 4°C. Then we measured enzyme activity, and the highest enzyme activity of each enzyme was considered as 100%, and other values were recorded as a percentage of this control.

### Measurement of kinetic parameters of various synthetic peptides

A total of three synthetic *p*‐nitroanilide (*p*NA) peptide substrates were used, namely AAPF (succinyl‐Ala‐Ala‐Pro‐Phe‐*p*NA), AAPL (succinyl‐Ala‐Ala‐Pro‐Leu‐*p*NA) and AAVA (succinyl‐Ala‐Ala‐Val‐Ala‐*p*NA), and kinetic parameters of the synthetic peptides were determined as previously described by Windhorst and colleagues (2002). Enzyme reactions with synthetic peptides were conducted in 5% v/v dimethylformamide in 100 mM Tris‐HCl buffer (pH 8.2) at 25°C. The enzyme concentration varied between 1.5 × 10^−8^ and 9.5 × 10^−9^ M, and the substrate was in the range of 0.1–1.6 mM. The release of *p*‐nitroaniline was measured at 405 nm (ε_405_ = 9600 M^−1^ cm^−1^) within initial time intervals by using a UV2450 spectrophotometer (Shimadzu, Japan). The values of the kinetic parameters *K_m_* and *k_cat_* were obtained from non‐linear regression analysis by using the uvprobe software (Shimadzu). The various assays were repeated at least twice, and the standard deviation was calculated.

### Thermal denaturation and CD spectroscopy

An MOS‐450 CD spectropolarimeter (Bio‐Logic, France) was used to record molar ellipticity (mdeg) to predict secondary structure, and a TCU‐250 auto‐titrator (Bio‐Logic) was used for thermal denaturation assays. The various keratinases (1–3 μM) were dissolved in 10 mM phosphate buffer (pH 7.0). The thermal denaturation curves of the proteins were obtained by plotting the changes in the molar ellipticity values at 222 nm against temperature, which increased by 1°C min^−1^. The thermal denaturation curves were normalized. The initial CD value was regarded as 0% unfolded, and the last CD value after heating at 80°C was considered as 100% unfolded. The midpoints of curves were calculated and regarded as *Tm* values. All experiments were repeated three times and the standard deviation was calculated.

Purified proteins were dissolved in 10 mM phosphate buffer (pH 7.0) at concentrations ranging from 20 to 100 μg ml^−1^. A 1 mm path length cuvette was used and spectra were recorded from 190 to 250 nm with a response time of 1 s and an accumulation of three scans. The DichroWeb CD analysis tool (http://dichroweb.cryst.bbk.ac.uk/html/home.shtml) was used for graphical analyses and the calculation of secondary structure.

### Homology modelling of different mutants and molecular dynamic (MD) simulations

Since the N‐propeptides showed low homology (< 20%) with other protein structures in the PDB database, they were not considered for modelling. The homology models of mature forms of KerSMD and mutant were constructed using the modeller v9.11 programme (www.salilab.org/modeller). The model of the catalytic domain C2 was based on several 3D structures of subtilisin‐like proteases (PDB no. 3LPA, 3LPC and 3LPD, with 47% identity), proteases BprB and BprV (PDB no. 3TI9 and 3TI7, with 47% identity), thermostable serine protease (PDB no. 1DBI, with 37% identity), thermitase (PDB no. 1THM, with 35% identity), and *Thermococcus kodakaraensis* protease (PDB no. 3AFG, about 25% identity). Models of the PPC domains of KerSMD (T2) and KerSMF (T1) were based on the templates of vEP C‐ter 100 (PDB no. 2LUW, with 30–50% identity) and kexin (PDB no. 1OT5, with 25% identity). The model with the lowest score was chosen, and then the C2 and PPC domains (T2 or T1) were linked together to obtain the models of DDD and DDF.

The MD simulations were performed to fully reduce steric clashes, using the namd software (www.ks.uiuc.edu/Research/namd/). The process used CHARMM force fields (www.charmm.org), periodic boundary conditions of a water box and the particle mesh Ewald algorithm. Using a constant temperature of 310 K, 1 atm pressure and 1 ns running time, minimum energy models were obtained. The procheck and errat programmes of the Structural Analysis and Verification Server (http://services.mbi.ucla.edu/SAVES/) were used to examine the stereochemical quality, showing favoured values for DDD and DDF of 95% and 94.2% respectively.

## Supporting information


**Fig. S1.** Amino acid sequences alignment and predicted second structure of keratinases KerSMD and KerSMF.
**Fig. S2.** Linear fitting of temperature kinetics of various keratinase mutants and wild type at 60°C.
**Fig. S3.** Effects of pH on enzyme activity and stability of KerSMD (P2C2T2) and different mutants.
**Fig. S4.** Effects of exogenous N‐propeptides on the activity of various proteins.
**Table S1.** Thermal stabilities of the wild type (DDD) and various mutants.
**Table S2.** Predicted secondary structure content using CD spectra data.
