# Supplementary material for: Enhancement of the catalytic efficiency and thermostability of S tenotrophomonas sp. keratinase KerSMD by domain exchange with KerSMF
Source: Microb Biotechnol. 2015 Nov 10;9(1):35–46. doi: 10.1111/1751-7915.12300 (PMC4720410; doi:10.1111/1751-7915.12300)
Supplement: Supplementary file 1 — Fig. S1. Amino acid sequences alignment and predicted second structure of keratinases KerSMD and KerSMF. Fig. S2. Linear fitting of temperature kinetics of various keratinase mutants and wild type at 60°C. Fig. S3. Effects of pH on enzyme activity and stability of KerSMD (P2C2T2) and different mutants. Fig. S4. Effects of exogenous N‐propeptides on the activity of various proteins. Table S1. Thermal stabilities of the wild type (DDD) and various mutants. Table S2. Predicted secondary structure content using CD spectra data. [file MBT2-9-035-s001.doc]

**Enhancement of the** **catalytic efﬁciency and thermostability of** ***Stenotrophomonas* sp. keratinase KerSMD by replacing a new N-propeptide or C-terminal domain from KerSMF**

**Zhen Fang** ^1, 2, 5^**, Juan Zhang** ^1^, **^5^ *, Baihong Liu**^1, 2, 5^**, Guocheng Du**^3^**^, 5^*****, Jian Chen ^4^,** ^5^

***^1^*** *Key Laboratory of Industrial Biotechnology, Ministry of Education, Jiangnan University, Wuxi 214122, China*

*^2^* *Synergetic Innovation Center of Food Safety and Nutrition, Wuxi 214122, China*

***^3^*** *The Key Laboratory of Carbohydrate Chemistry and Biotechnology, Ministry of Education, Jiangnan University, Wuxi 214122, China*

***^4^*** *National Engineering Laboratory for Cereal Fermentation Technology, Jiangnan University, Wuxi 214122, China*

*^5^* *School of Biotechnology, Jiangnan University, Wuxi 214122, China*

*Corresponding authors: Juan Zhang, E-mail: [zhangj@jiangnan.edu.cn](mailto:zhangj@jiangnan.edu.cn), Tel.: +86-510-85918307, Fax: +86-510-85918309.

Guocheng Du, E-mail: gcdu@jiangnan.edu.cn. Tel./Fax: +86-510-85918309.

School of Biotechnology, Jiangnan University, 1800 Lihu Road, Wuxi 214122, China.

**Fig. S1** Amino acid sequences alignment (A) and predicted second structure (B) of keratinases KerSMD and KerSMF. A:Yellow area is the N-propeptide. Asterisk means the identity of amino acid, colon and point mean the similarity of amino acid. B: Blue arrow means the fold; red stick means the helix; gray line means the loop.

**A**

KerSMD AGLPTREPVRQASTAEPGAERIIVKYRAGTAAAGDRSAKLSTVQSALTRASLSGGTARAS 60

KerSMF -----GDVQLSGLQSAPTHQRFIVKYRDGSAPVANTTALASSLKSAAAGLASSQGRA--- 52

: .. : * :*:***** *:*...: :* *:::** : : * * *

KerSMD TLGPQVVRKLGVGADLIRLQGRLAPAELQRVLKELKADPAVQYAEADVKLRRTELRAGDV 120

KerSMF -LGLQEVRKLAVGPTLVRTDRPLDQAESELLMRKLAADPNVEYVEVDQIMR--------- 102

** * ****.**. *:* : * ** : :::: * *** *:*.*.* :*

KerSMD QPALAPNDPYYQQYQWHLHNATGGINAPSAWDVSQGEGVVVAVLDTGILPQHPDLVGNLL 180

KerSMF -ATLTPNDTRFSE-QWGFGTSNASINVQPAWDKATGTGVVMAVIDTGIT-NHPDLNANIL 159

.:*:***. :.: ** : .:...**. . *** : * ***:**:**** :**** .*:*

KerSMD EGYDFISDAETSRRATNDRVPGAQDYGDWVENDNECYTGSVAEDSSWHGTHVAGTVAEQT 240

KerSMF PGYDFISDAAMARDGG-GRDNNPNDEGDWYG-ANECGSGIPASNSSWHGTHVAGTVAAVT 217

******** : * . .* ..:* *** *** :* *.: ************* *

KerSMD NNGVGMAGVAHKAKVLPVRVLGKCGGYLSDIADAITWASGGTVAGVPANANPAEIINMSL 300

KerSMF NNSTGVAGTAFNAKVVPVRVLGKCGGYTSDIADAIVWASGGTVSGVPANANPAEVINMSL 277

**..*:**.*.:***:*********** *******.*******:**********:*****

KerSMD GGSGSCDGTYQDAINGAISRGTTVVVAAGNETDNASKYRPASCDGVVTVGATRITGGITY 360

KerSMF GGGGSCSTTYQNAINGAVSRGTTVVVAAGNSNTNVSSSVPANCPNVIAVAATTSAGARAS 337

**.***. ***:*****:************.. *.*. **.* .*::*.** : *. :

KerSMD YSNYGTRVDLSGPGGGGSVDGNPGGYVWQSGSDAATTPESGSYSYMGMGGTSMASPHVAA 420

KerSMF FSNYGTGIDISAPGQS----------ILSTLNTGTTTP--GSASYASYNGTSMAAPHVAG 385

:***** :*:*.** . : .: . .:*** ** ** . .*****:****.

KerSMD VAALVQSALIAKGKDPLAPAAMRTLLKETARPFPVSIPAATPIGTGIVDAKAALAKALEE 480

KerSMF VVALMQSVAPS----PLSPAQVESIIKSTARPLPGACSGG--CGAGIVDANAAVAAAING 439

*.**:**. : **:** :.:::*.****:* : ... *:*****:**:* *::

KerSMD PCTESCGPVATPLTNKAAVGGLNGTAGSSRLYSFEAAAG-KQLSVITYGGTGNVSVYIAQ 539

KerSMF GGNPNPG--GNVLQNNVPVTGLGAATGAELNYTVAVPAGSSQLRVTISGGSGDADLYVRQ 497

. . * .. * *:.. * **..::*:. *:. .. ** .** * **:*:..:*:*

KerSMD GREPSASDNDGKSTRPGTSETVRVNKPVAGTYYIKVVGEAAYNGVSILATQ 590

KerSMF GSAPTDTSYTCRPYLSGNSETCTINSPAAGTWYVRVKAYSTFSGVTLNAQY 548

* *: :. :. .*.*** :*.*.***:*::* . :::.**:: *

**B**

T2 of KerSMD:


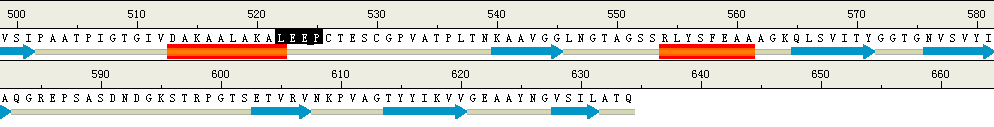


T1of KerSMF:


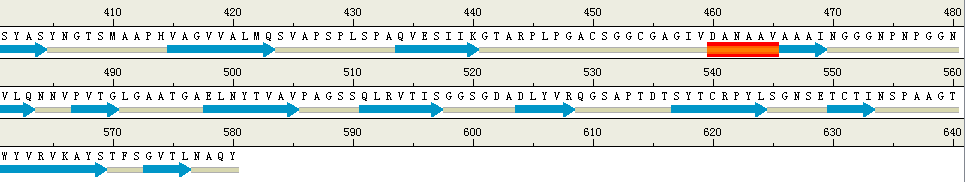


**Table S1.** Thermal stabilities of the wild type (DDD) and various mutants. The *T_m_* value is obtained by calculating the midpoint value of the thermal denaturation curves, which is based on a least-squares analysis. The △*T_m_* value is the result of increased or decreased *T_m_* value comparing to KerSMD. The half-life period (*t_1/2_*) was calculated from the changes of residual activity along with time course at 60 °C incubation.

| Protein | *T_m_* (°C) | △*T_m_* (°C) | *t_1/2_* (min) |
| --- | --- | --- | --- |
| DDD | 56.00±0.48 | -- | 41±15 |
| DDF | 62.50±0.46 | +6.50 | 105.3±5 |
| FDD | 63.20±0.48 | +7.20 | 152.2±2 |
| FDF | 64.60±0.65 | +8.60 | 244.6±2 |
| DD | 47.80±0.88 | -8.20 | 5.6±2 |
| FD | 60.80±0.93 | +4.80 | 36.5±6 |

**Table S2.** The predicted secondary structure content using CD spectra data.

| Enzyme | CD secondary structure content (%) | | | |
| --- | --- | --- | --- | --- |
|  | *α*-Helix | *β*-Sheet | Turns | Unordered |
| DDD | 10.5 | 37.1 | 19.1 | 29.0 |
| DDF | 18.6 | 33.0 | 21.0 | 27.1 |
| FDD | 12.0 | 42.3 | 15.5 | 21.2 |
| FDF | 19.3 | 30.7 | 20.9 | 28.5 |
| DD | 14.2 | 47.2 | 10.4 | 32.9 |
| FD | 9.1 | 27.5 | 23.9 | 38.1 |

**Fig. S2** The linear fitting of temperature kinetics of various keratinase mutants and wild type at 60 ℃. P2C2T2 represents DDD; P2C2T1 represents DDF; P1C2T2 represents FDD; P1C2T1 represents FDF; P2C2 represents DD; and P1C2 represents FD.


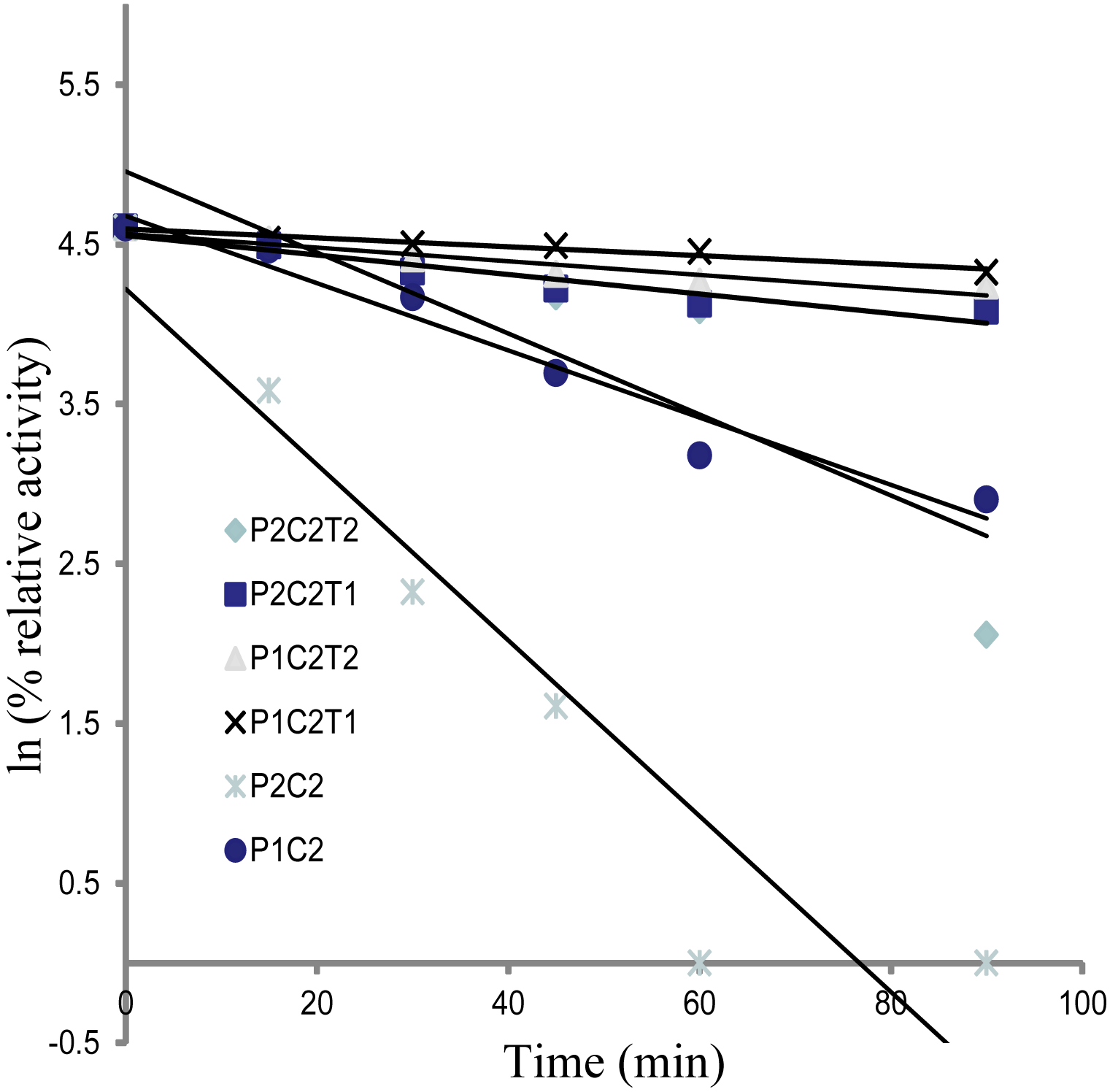


**Fig. S3.** Effects of pH on enzyme activity and stability of KerSMD (P2C2T2) and different mutants. P2C2T2 represents DDD; P2C2T1 represents DDF; P1C2T2 represents FDD; P1C2T1 represents FDF; P2C2 represents DD; and P1C2 represents FD.

A


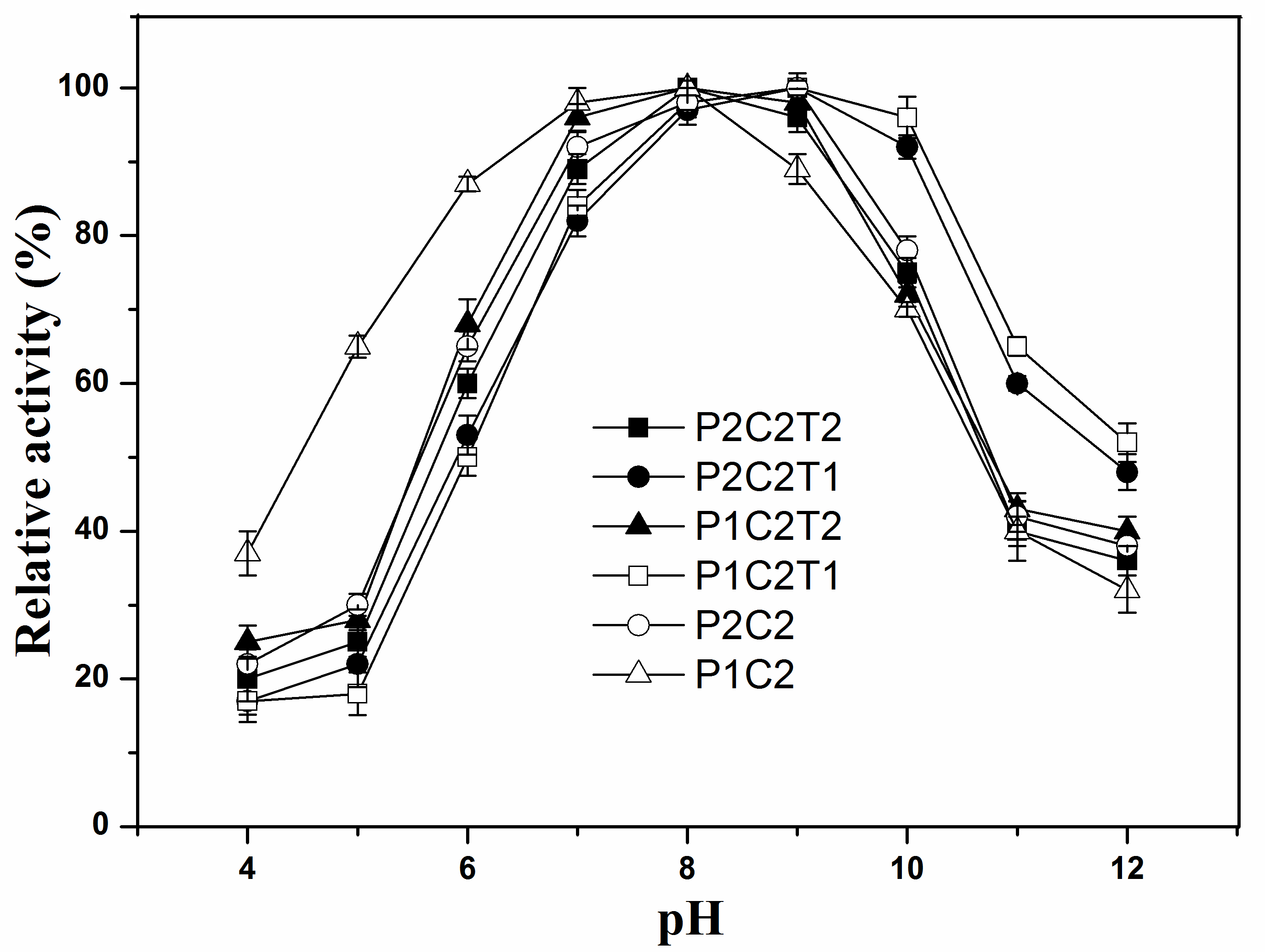


B


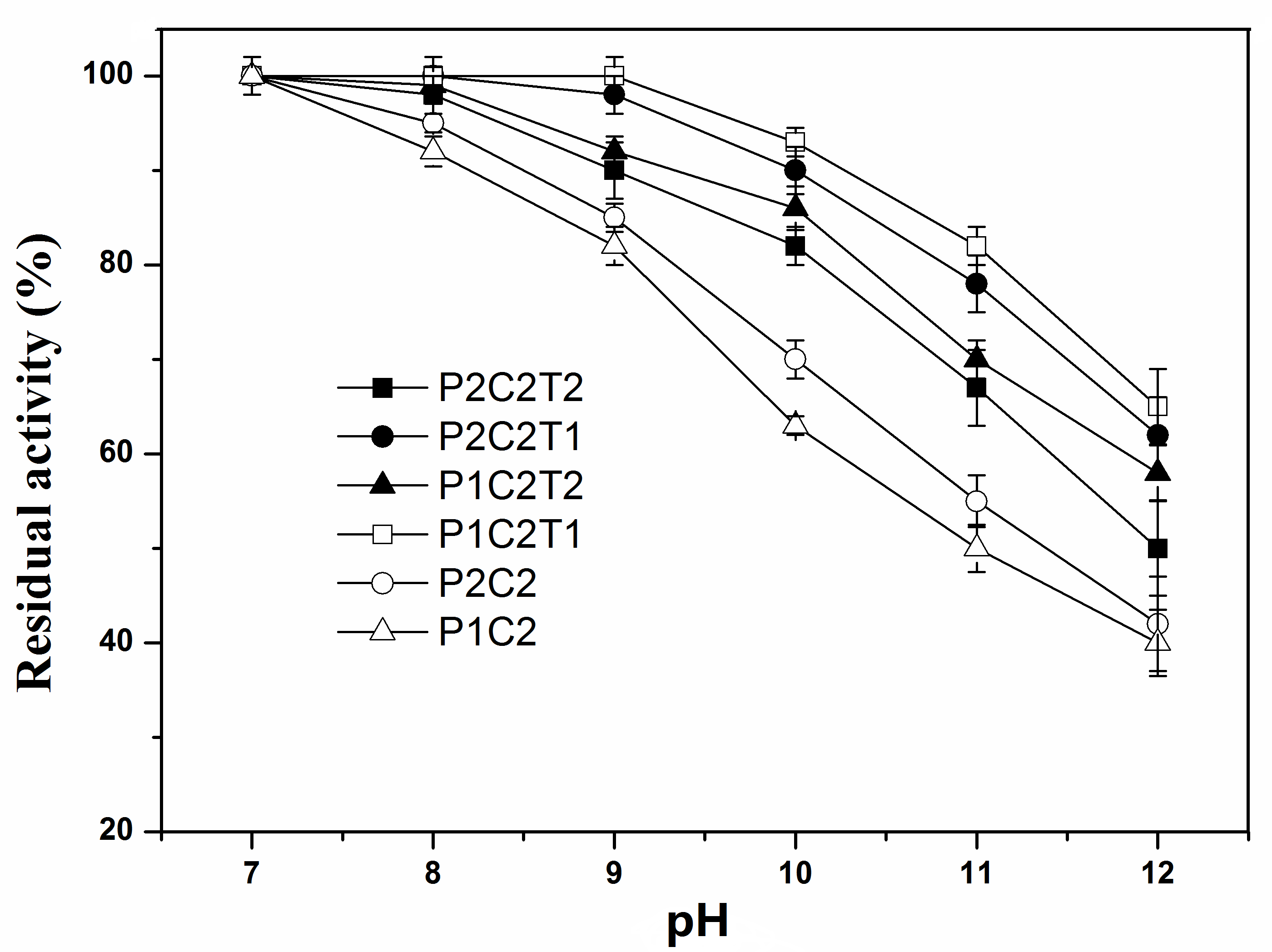


**Fig. S4.** The effects of exogenous N-propeptides on the activity of various proteins. Purified proteins was mixed with same molar concentration (0.02 mg/ml) of BSA or N-propeptides. The addition of BSA was treated as a control and regarded as 100% activity, except the inactive samples C1T1 and C2T2 was regarded as zero activity.

A


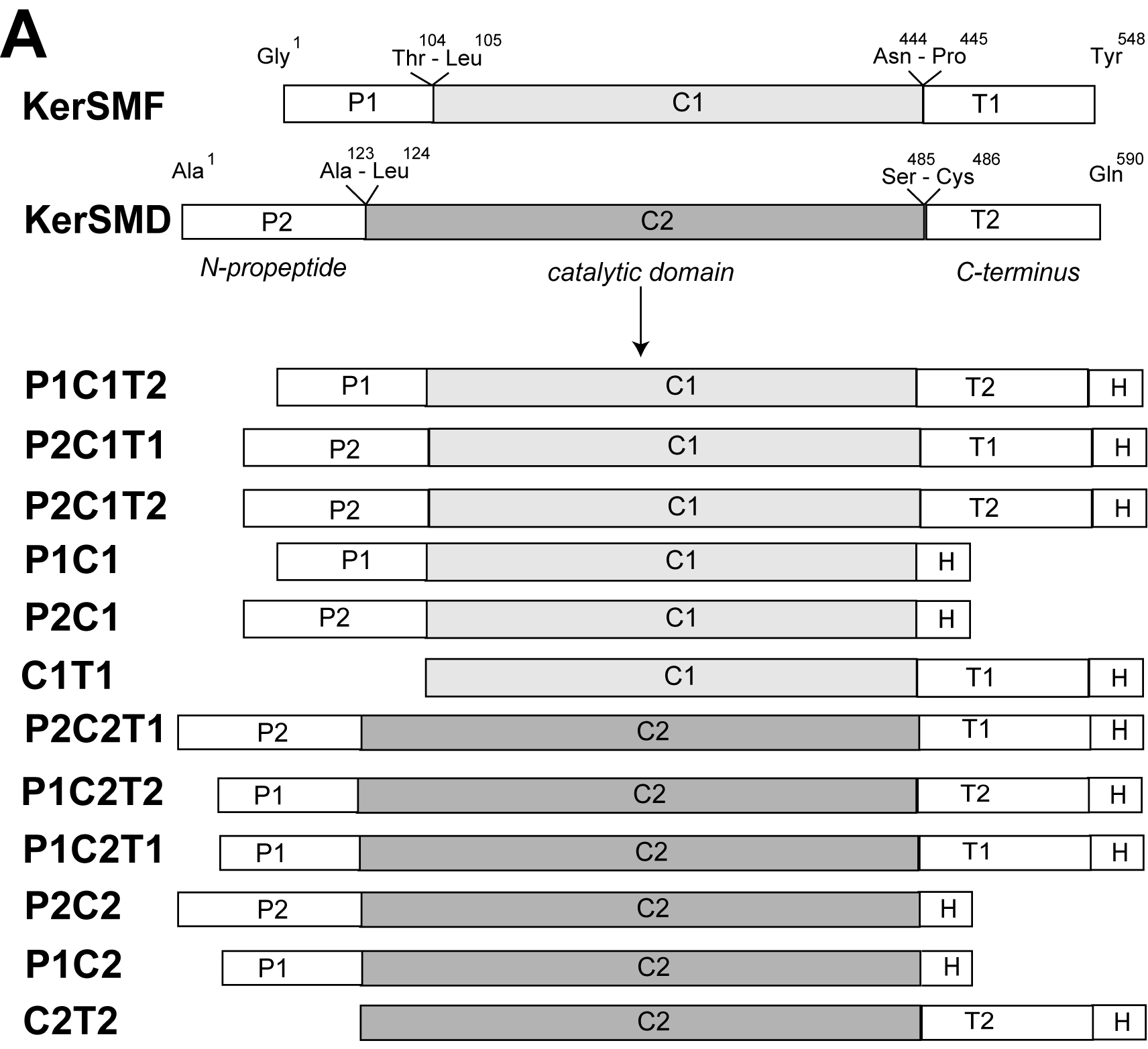


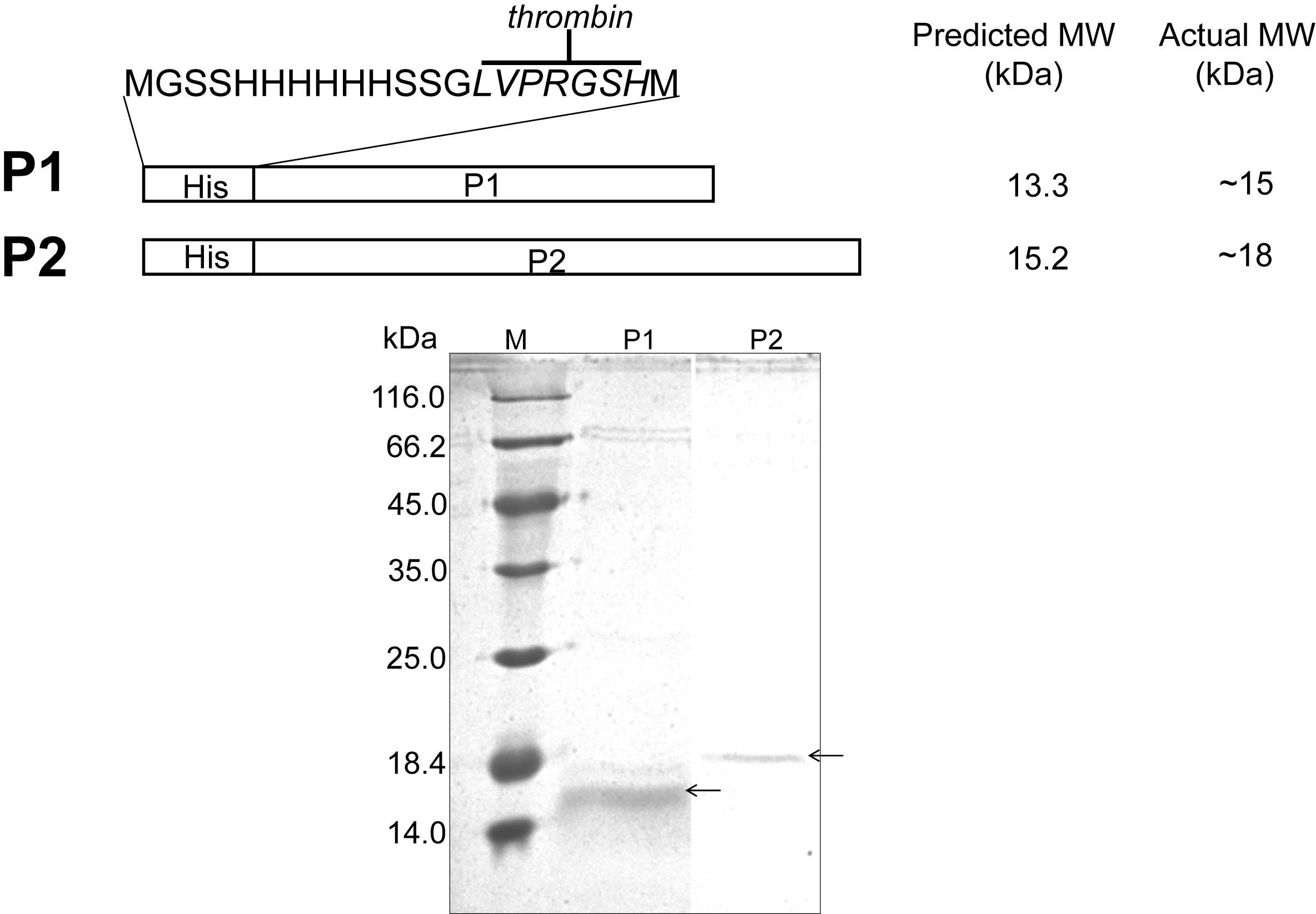


B


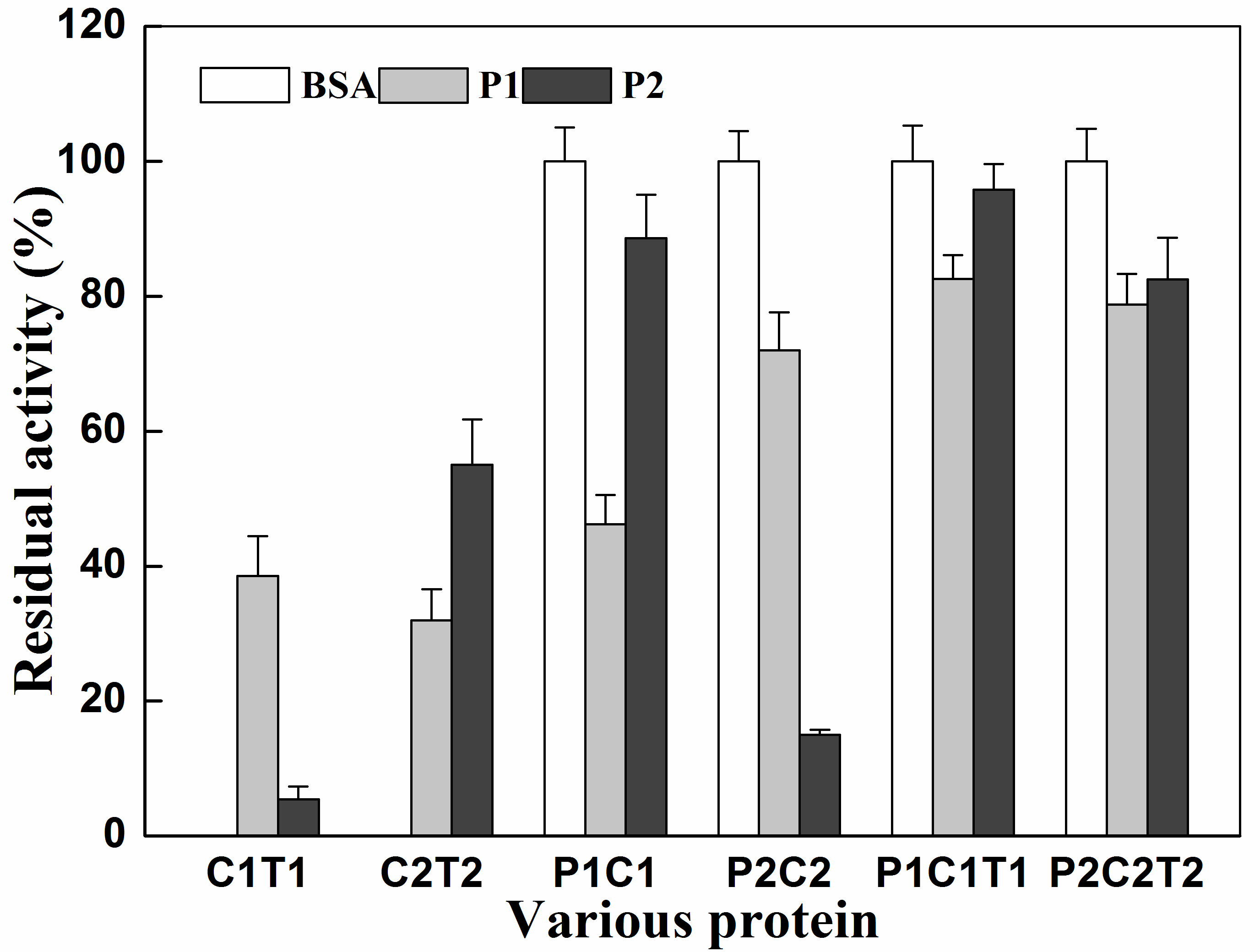


C


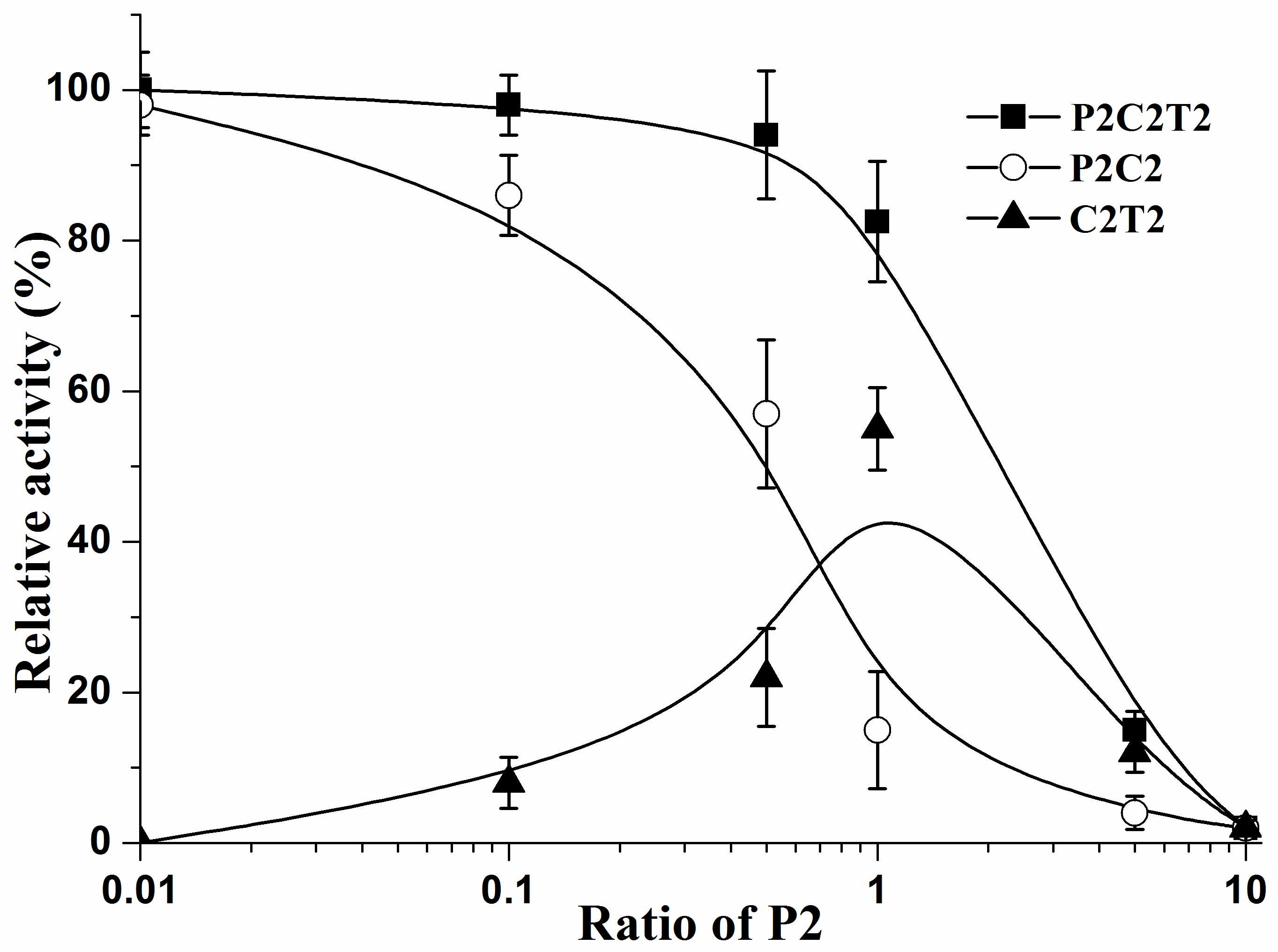


Effects of exogenous N-propeptides on enzyme activity

To investigate whether N-terminal propeptide could affect enzyme activity or had the function to refold error protein, it was exogenously added to the inactive proteins C1T1, C2T2, and P2C1, the wile type enzymes P1C1T1 and P2C2T2, and the C-terminal deletion mutants P2C2 and P1C1. The N-propeptides of KerSMD and KerSMF were expressed and purified individually. The primal N-propeptides with N-terminal His-tag were treated with thrombin to cut off His-tag. The results showed that noncovalently linked N-propeptides P1 and P2 all had the different ability to inhibit enzyme activity. The wild type enzymes P1C1T1 and P2C2T2 dropped small partial protease activity when P1 or P2 was added. P1C1 and P2C2 were also inhibited by exogenous N-propeptides, showing more obvious inhibition than P1C1T1 and P2C2T2. On the other hand, it was showed that N-propeptides could activate C1T1 and C2T2 by protein renaturation *in vitro*. As shown in Fig. S4, the P2 was feeble to refold active C1T1 but greatly increase C2T2 activity. And the optimal ratio of molar concentration was 1:1 for P2 to refold C2T2. With the increase of P2 concentration, protease activity of the renaturation C2T2 dropped down, which was the similar case to P2C2 and P2C2T2. Higher concentration of P2 was needed to inhibit the activity of P2C2T2. It seemed that mature protein of P2C2T2 has at least two substrate-binding pockets: one is catalytic center and another is non-catalytic pocket. The inactive mutant P2C1, which had N-propeptide in its mature form, could not be refold into active enzymes by exogenous N-propeptide P1 or P2 (data not show). The covalently linked P2 might inhibit protease activity of C1.
